# Antimicrobial peptide capsids of de novo design

**DOI:** 10.1038/s41467-017-02475-3

**Published:** 2017-12-22

**Authors:** Emiliana De Santis, Hasan Alkassem, Baptiste Lamarre, Nilofar Faruqui, Angelo Bella, James E. Noble, Nicola Micale, Santanu Ray, Jonathan R. Burns, Alexander R. Yon, Bart W. Hoogenboom, Maxim G. Ryadnov

**Affiliations:** 1 National Physical Laboratory, Hampton Road, Teddington, TW11 0LW UK; 2 London Centre for Nanotechnology, University College London, London, WC1H 0AH UK; 3 Department of Biochemical Engineering, University College London, London, WC1E 6BT UK; 4 Dipartimento di Scienze Chimiche, Biologiche, Farmaceutiche ed Ambientali, Università degli Studi di Messina, 98168 Messina, Italy; 5 SET, University of Brighton, Brighton, BN2 4GJ UK; 6 Department of Chemistry, University College London, London, WC1E 6BT UK; 7 Institute of Structural and Molecular Biology, University College London, London, WC1E 6BT UK; 8 Department of Physics & Astronomy, University College London, London, WC1E 6BT UK

## Abstract

The spread of bacterial resistance to antibiotics poses the need for antimicrobial discovery. With traditional search paradigms being exhausted, approaches that are altogether different from antibiotics may offer promising and creative solutions. Here, we introduce a de novo peptide topology that—by emulating the virus architecture—assembles into discrete antimicrobial capsids. Using the combination of high-resolution and real-time imaging, we demonstrate that these artificial capsids assemble as 20-nm hollow shells that attack bacterial membranes and upon landing on phospholipid bilayers instantaneously (seconds) convert into rapidly expanding pores causing membrane lysis (minutes). The designed capsids show broad antimicrobial activities, thus executing one primary function—they destroy bacteria on contact.

## Abstract (web summary)

With the growing threat of antibiotic resistance, unconventional approaches to antimicrobial discovery are needed. Here, the authors present a peptide topology that mimics virus architecture and assembles into antimicrobial capsids that disrupt bacterial membranes upon contact.

## Introduction

Antibiotic resistance presents significant challenges for healthcare^1^. Conventional antimicrobial strategies that target individual intracellular processes continue facing the same barriers of rapidly acquired resistance^2^, impermeable bacterial membranes^3^ and the tolerance of quiescent bacteria to antibiotic treatments^4^. These are just a few factors that prompt the development of novel antimicrobial agents that would be compatible with mammalian immune systems, would not differentiate between growing and dormant bacteria and whose activity does not rely on reaching intracellular targets. In this regard, host defense peptides provide a promising source of antimicrobial agents^5^. These molecules are evolutionarily conserved components of the innate immunity that are found in all living organisms. Despite their apparent diversity, the peptides share common characteristics. They fold into amphipathic structures in response to membrane binding, are cationic and cause disrupting pores, channels or carpets in microbial phospholipid bilayers^6^. To realise these modes of action, the peptides must translate from an inactive surface-bound (“S”) state to an active and antimicrobial pore-like insertion (“I”) state^7^. This transition necessitates the localised oligomerisation of individual peptides in membranes that would be able to overcome a threshold barrier—a critical local concentration above which antimicrobial effects become apparent. Removing this barrier by locally delivering concentrations that greatly exceed the threshold concentrations may introduce strategies with potentially faster and more profound antimicrobial responses that are less subject to acquired resistance.

Here, we show that artificial virus-like capsids assembled from de novo antimicrobial peptide sequences provide such a strategy. These designed capsids assemble as 20-nm hollow shells that favour attack on bacterial membranes and upon landing on phospholipid bilayers instantaneously convert into rapidly expanding pores causing bacterial lysis within minutes. We demonstrate that these capsids exhibit broad antimicrobial activities, thus executing one primary biological function. They destroy bacteria on contact.

## Results

### Capsid design principles

Our approach is a retro design that aims to encode a pore-forming oligomerisation state into a discrete pre-assembly of antimicrobial peptides. Since membrane-disrupting pores need not exceed tens of nanometres, the resulting assembly has to be structurally confined to avoid aggregation, an order of magnitude smaller than a bacterial cell (0.2–2 µm) to instantaneously convert into membrane-lysing pores, and functionally inert (non-toxic) to mammalian cells. The viral capsid architecture offers a suitable self-assembly form that is symbiotic to mammalian host cells and can meet all of these requirements. Viruses do not disrupt microbial membranes, are not typically antimicrobial, but their capsids offer an ideal, amenable architectural platform for designing artificial capsids assembled from antimicrobial sequences. Such synthetic capsids have a primary biological function of destroying bacterial membranes on contact.

**Fig. 1:**
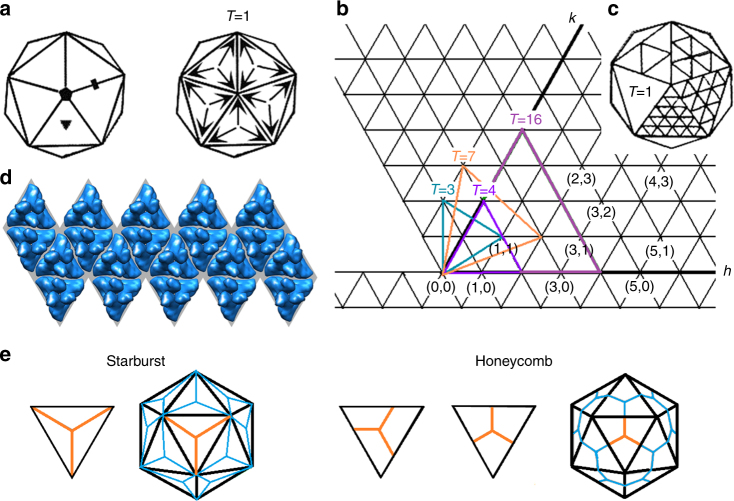
The geometry of viral capsids. **a** Schematic representation of an equilateral icosahedron; 20 flat triangular faces and 12 vertices are arranged around a sphere and related by five-, three- and two-fold axes of rotational symmetry (diamond, triangle and rectangle, respectively). With each triangular face hosting three protein subunits (arrows at the vertices), 60 equivalent subunits in total, the icosahedron becomes a *T* = 1 capsid. **b** Equilateral triangular net relating icosahedral and quasi-equivalent symmetries. Each triangulation (*T*) number is calculated from (*h, k*) and denotes the number of smaller triangles arranged around local quasi-6-folds in one triangular icosahedron face. **c** The net folded into an icosahedron showing different *T* numbers in individual triangles: (clockwise) 1, 4, 9, 16 and 25. **d** Native virus (*T* = 1) capsid unfolded into a triangular net with three identical subunits in each triangular face. The figure (Vdb 898, PDB 2GSY) was obtained from VIPERdb (http://viperdb.scripps.edu)^9^. **e** Two arrangements of a *C*_3_-triskelion within a triangular unit leading to “starburst” and “honeycomb” propagation modes. In the starburst mode, the three equivalent legs of the triskelion serve as the altitudes of the triangle. Two variations of the honeycomb triskelion arrangement, symmetrical and asymmetrical, are given. For clarity, central triskelions are shown in orange

Native capsid proteins assemble according to an *n*-fold rotational symmetry, which imposes geometric constraints on allowed protein folds and interactions. Often it means that more protein subunits or subunit proteins are used in a capsid. Any capsid can be described using the concept of quasi-equivalent positions. The concept offers an approach whereby capsid proteins are packed around the surface of a sphere with a cubic symmetry, may be positioned in similar environments (quasi-equivalent) with respect to each other, and are clustered into closed supramolecular networks^8^. These principles are universal and pertinent to viruses from different taxonomic groups: human (adenoviruses), plant (cowpea mosaic viruses), animal (porcine parvovirus), algal (phycodnavirus) or bacterial (tailless phage) capsids are all proteinaceous icosahedra^9^. An icosahedron consists of 20 flat faces of equilateral triangles arranged around a sphere with 5/2 3 symmetry (Fig. 1a).

Each face is subdivided into smaller triangles, facets, and their number is reflected in a triangulation number *T*, which can be found graphically from an equilateral triangular net and using a simple equation:

1 $$T = k^2 + k \times h + h^2,$$

where *k* and *h* are any integers with no common factor (Fig. 1b).

Capsids with low triangulation numbers would have low numbers of different polypeptide sequences and subunits in the assembly (Fig. 1c). Indeed, a *T* = 1 capsid has 20 triangular faces and each triangle can be subdivided into three equal portions each hosting one protein (Fig. 1a). A total of 60 identical proteins would make up a *T* = 1 capsid (Fig. 1d). Any *T *> 1 icosahedron has 20 × *T* faces, 12 pentamers (five-fold vertices) and 10 × (*T–*1) hexamers. As a consequence, protein numbers in capsids increase with increasing *T* numbers, e.g. *T *= 3 (180 proteins), *T *= 4 (240) and so on. The capsid symmetry is ensured by the arrangement of protein subunits around five- and six-fold vertices into pentameric and hexameric supramolecular units, respectively (Fig. 1b). Capsid proteins interface via precisely matched orthogonal interactions^10^, which remain challenging to emulate de novo.

To mitigate the need for such orthogonal fitting, the same protein motif can be placed at each triangular vertex, whereas each triangle must retain the three-fold symmetry (Fig. 1a). To accommodate this requirement, we designed a rigid *C*_3_-triskelion to occupy a triangle (Fig. 1e). Two triskelion arrangements within a triangle are possible, leading to two different propagation modes, “starburst” and “honeycomb” (Fig. 1e). Each adapts a classical 5/2 3 capsid symmetry, and each requires the triskelion to propagate at its termini. However, the “honeycomb” propagation, albeit successfully exploited for the design of peptide shells, relies on interfacial “sticky” interactions, oblique or orthogonal^11–17^. These use naturally occurring peptides, typically forming β-pleated structures, that assemble into amyloid-like aggregates^11,15–17^, and artificial helices whose assembly depends fully on stabilisation via disulphide bridging^13^ or benefits from it^12,14^. Three-arm dendrimers or three-directional, non-covalent branching hubs in this mode propagate at two-fold axes of symmetry, with agglomeration and fibrillogenesis being common side reactions^13–15^. Strong binding at two-fold axes is crucial as the five- and six-fold vertices are deemed largely hollow and redundant^18^. Therefore, only short triskelions or orthogonal *C*_3_-branching can afford tight packing supporting this mode of propagation. By contrast, the “starburst” arrangement is better suited to direct a capsid-like assembly. It is enabled by all three *C*_3_-termini clustering at five- and six-fold axes and can readily translate into any *T* > 1 assembly. The three equivalent legs of each triskelion provide the three altitudes of a triangle. Therefore, the edge of a triangular unit is a common integer for all units in an icosahedron face, irrespective of their number (Fig. 1b, c). Based on these principles, we engineered an antimicrobial protein motif arranged into a *C*_3_-triskelion that by clustering at its leg termini with other triskelion copies assembles into capsid-like shells.

### An elementary *C*_1_-subunit

To provide a triangular unit, each leg of a triskelion must be a rigid, rod-like structure. A protein folding motif to provide this property is an α-helical coiled coil^19^. The motif is ubiquitous for different viral processes ranging from capsid assembly (hepatitis B virus) to membrane fusion (HIV)^20,21^. Coiled coils are bundles of interdigitated α-helices, the number of which can be rationally controlled. In viruses, however, the motif is predominantly heterodimeric and packs further forming four- and six-helix bundles^20–22^. Canonical coiled coils are characterised by heptad repeats of hydrophobic and polar residues, typically designated *abcdefg*, in which *a* and *d* form hydrophobic interfaces^23^. In order to form contiguous interfaces, the *a*/*d* patterns of corresponding helices must be in register. Yet, the average spacing of hydrophobic residues along a coiled-coil sequence is 3.5 residues, which is less than one complete turn (3.6 residues) of a monomeric α-helix^19^. To rectify this discrepancy, *a*/*d* patterns impose a slight left-handed twist, enabling left-handed helix-crossing angles in the coiled-coil bundle. This permits stable coiled-coil formation. Complementary electrostatic interactions at *e* and *g* sites of successive heptads between partner strands (i.e.* g*–*e*′ interactions: *g* of one heptad and *e*′ of the following heptad on the other helix) stabilise the coiled coil^24^. Recently, we have expanded the assignment of coiled-coil patterns into naturally occurring and de novo antimicrobial sequences^25–27^.

**Fig. 2:**
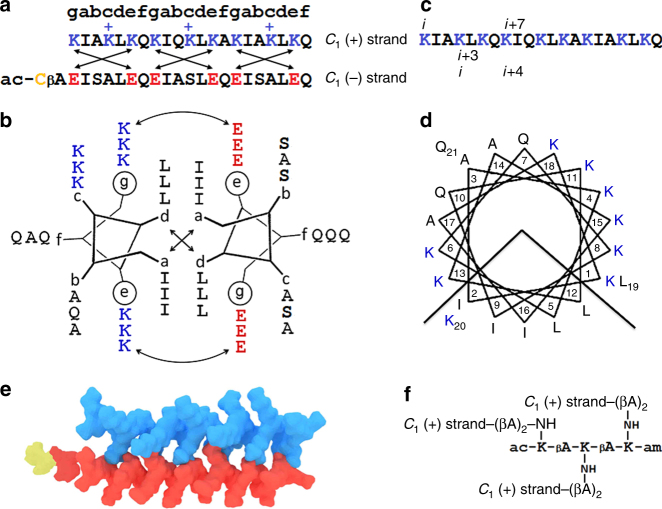
An elementary capsid *C*_1_-subunit. **a** The linear sequences of the subunit. Antimicrobial (*C*_1_ (+) strand) and antagonist (*C*_1_ (−) strand) sequences are shown in blue and red, respectively, and are aligned under the coiled-coil heptad repeat pattern, *gabcdef*. The cysteine residue is shown in yellow. Crossed arrows show electrostatic interactions. Unengaged positively charged lysines are highlighted with “+”. These confer the dimer with an overall positive charge facilitating the binding of the subunit to anionic microbial membranes (see also Supplementary Table 1). **b** The sequences configured onto a coiled-coil helical wheel with 3.5 residues per turn. Curved double-headed arrows indicate electrostatic interactions between *g* and *e*′ (circled) residues. Crossed arrows show the hydrophobic interface of the dimeric subunit formed by isoleucines and leucines in *a* and *d*, respectively. **c** The linear sequence of the *C*_1_ (+) strand. Monomeric (*i, i* + 7) and coiled-coil (*i, i* + 3 and *i, i* + 4) helical spacings are shown, one of each for clarity. **d** The sequence of the *C*_1_ (+) strand configured onto a monomeric helical wheel with 3.6 residues per turn, showing the clustering of amino-acid residues into two distinctive polar and hydrophobic faces. **e** Molecular model of the coiled-coil *C*_1_-subunit (PDB entry 4DMD, rendered by Macromodel Schrödinger). **f** The chemical structure of a dendrimer *C*_3_-hub used to make the *C*_3_-triskelion, *C*_3_ (+) strand. βA denotes beta-alanine (see also Supplementary Fig 1)

For engineering antimicrobial coiled coils, it is necessary to consider hydrophobic *a*/*d* pairs in conjunction with charged *g*/*e*′ pairs^25^. In this case, a coiled-coil heterodimer would have an antimicrobial cationic strand and a complementary anionic strand. To enable such a dimer, we made *a*/*d* pairs isoleucine/leucine, thus emulating native leucine-zipper structures, while cationic lysines (antimicrobial strand) and anionic glutamates (antagonistic strand) were used for complementary *g*/*e*′ pairs supporting parallel heterodimers. Additional lysine residues were introduced into the solvent-exposed *c* sites of the antimicrobial strand to facilitate coiled-coil disassembly upon binding to microbial membranes. The other solvent-exposed positions, *b* and *f*, were occupied by alternating neutral polar glutamines and small alanines. In the anionic strand, serine residues alternating with alanines in *b* and *c* positions were used to promote peptide solubility, with *f* sites taken up by glutamines. Given the minimum requirement of three contiguous heptads to form stable coiled coils^28^, and that antimicrobial sequences of similar lengths are appreciably strong^29^, each strand was made as a 21-mer (Fig. 2a–d, Supplementary Fig 1 and 2). This dimeric coiled coil offered an elementary geometric building block—an anisotropic, rod-shaped 3.2 × 2-nm *C*_1_-subunit (Fig. 2e).

Circular dichroism (CD) spectroscopy revealed that individually the peptides remain unfolded in solution, while helix formation was essentially complete (>90%) for their equimolar mixtures (Supplementary Fig 3a). The folding of the *C*_1_-subunit proved to be highly cooperative as gauged by nearly sigmoidal unfolding curves^30^ with a dominating transition midpoint (*T*_M_) of ~50 °C (Supplementary Fig 3b), and by the constant signal intensity at 202 nm during thermal denaturation, which provided a clear isodichroic point typical of a two-state, helix-to-coil, transition (Supplementary Fig 3c). The transition was also fully reversible as spectra recorded before and after the melt were nearly identical (Supplementary Fig 3d). As expected, no assemblies were observed for this elementary subunit by transmission electron microscopy (TEM) (Supplementary Fig 4a). For it to be placed at the triangular vertices and to endow it with a capsid-like symmetry, further modifications were necessary.

### A capsid *C*_3_-subunit

**Fig. 3:**
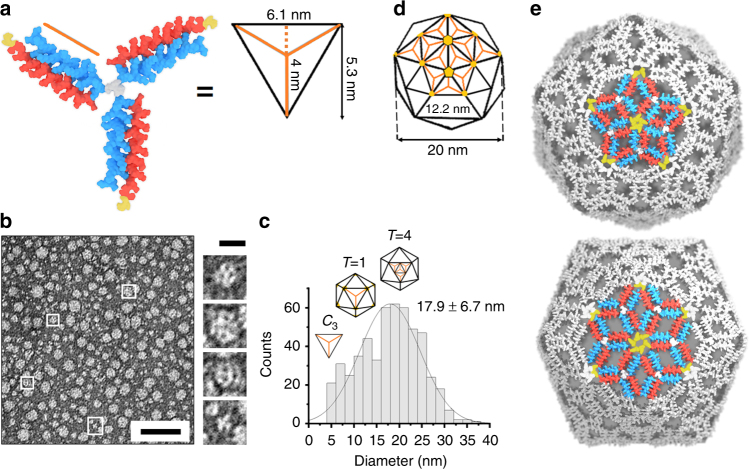
Capsid-like assembly of the *C*_3_-subunit. **a** Molecular model of the *C*_3_-triskelion (PDB entry 4DMD rendered by Macromodel Schrödinger). Each subunit of the triskelion corresponds to the altitude of an equilateral triangle. A folded subunit spans ~4 nm (three coiled-coil heptads extended with the arm of the dendrimer hub and the cysteine cap), which makes the side of the hosting triangle ~6.1 nm. **b** High-resolution electron micrographs for *C*_3_ (+) strand (100 µM) assembled with *C*_1_ (−) strand at 1:3 molar ratio, with individual structures highlighted by white squares. Scale bars are 100 nm for the large panel and 20 nm for the four inset images of individual assemblies. **c** Diameter distributions of the assembled capsids with corresponding sizes calculated for the triskelion fitted into a triangular facet (*C*_3_) and *T* capsids. **d** *T* = 4 icosahedron with four facets in each triangular face with each accommodating one triskelion. For clarity, only a partial network of triskelions in the icosahedron is shown. Five- and six-fold axes are highlighted by yellow diamonds. The diameter of the icosahedron and the side of its triangular face are calculated based on the triskelion dimensions shown in **a**. **e** Model of a *T* = 4 capsid assembled from the *C*_3_-subunit with parameters matching those from **a** and **d**. For clarity, only one of five- and six-fold axes each is shown in colour and with cysteines

First of all, three copies of the *C*_1_ (+) strand were conjugated onto a triskelial hub (Fig. 2f and Supplementary Fig 1). Each leg of the resulting structure folded with the *C*_1_ (–) strand giving rise to a rigid *C*_3_-triskelion (Fig. 3a).

All three legs are folded and identical, all carry a large positive net charge, which provides electrostatic repulsions, all have polar solvent-exposed faces with no complementary interactions and none can support anti-parallel coiled-coil formation. This rigid *C*_3_-subunit firmly fits into a triangular face, but cannot yet assemble. In accord with this, particulate structures with sizes of the subunit (5.3 ± 1.5 nm), and without higher order assemblies, were observed by TEM (Supplementary Fig 4b, c). Dynamic Light Scattering (DLS) provided complementary evidence by revealing an average hydrodynamic diameter of 5.25 ± 0.61 nm for the subunit in solution (Supplementary Fig 4d, e). Therefore, we set out to promote a directed aggregation of triskelions via their termini.

To enable this, the N-terminus of the *C*_1_ (−) strand was extended with a cysteine cap (Figs. 2a, e and 3a and Supplementary Fig 1). Cysteines, in both reduced and oxidised forms, have the highest hydrophobicity among the proteinogenic amino acids, possess high propensity for secondary structure and are capable of localised, proximity-driven cross-linking^14,31^. Buried, unpaired cysteines are known to induce particle formation in antibodies providing interfacial hydrophobic clustering accompanied by non-native cystine formation^32^. Similarly, viruses, in which native disulphide bonds are reduced or replaced, retain their ability to assemble into wild-type forms^33,34^. Collectively, these factors suggest that *C*_3_-oriented N-terminal cysteines of the triskelions should promote hydrophobic clustering at the vertices of triangular facets resulting in a capsid-like assembly. Consistent with this, DLS measurements gave average diameters of 21.2 ± 5.3 nm indicating the formation of higher order assemblies with a relatively narrow size distribution (Supplementary Fig 4f, g). It should be noted, however, that DLS is limited in size resolution by the factor of 3, which may not be sufficient to resolve complex and narrow distributions. Therefore, we sought more accurate evidence. The assembled capsids were analysed by high-resolution TEM, which showed broadly uniform paracrystalline phases with an average diameter of 18 nm (Fig. 3b). The importance of this finding is three fold.

Firstly, it supports a structural plasticity of the assembled capsids within a narrow size range. Such polymorphism is indeed common among viruses that can tune packaging symmetry to compensate for size variations^35^. As a rule, these changes are reflected in different *T* numbers^36^. For example, cowpea chlorotic mottle virus can repack from *T* = 3 (28 nm) into *T* = 1 (18 nm) capsids^37^. In our design, each leg of the folded triskelion (from the centre of the dendrimer hub to the acetylated N-terminus of *C*_1_ (−) strand) spans approximately 4 nm (Fig. 3a). This makes the side of an equilateral triangle ~6.1 nm, which falls within the sizes observed by TEM (Supplementary Fig 4b, c). A *T* = 1 assembly is deemed unstable and geometrically disfavoured as the triskelions cannot arrange around six-fold axes in this capsid (Fig. 1e and Supplementary Fig 5). Therefore, smaller assemblies observed by TEM are likely to be aberrant or incomplete capsids (Fig. 3b, c). Since the triskelions can only pack into equilateral triangular facets, the next possible assembly, in which triskelia termini cluster into clearly defined six- and five-fold symmetries, is a *T* = 4 capsid of ~20 nm in diameter (Fig. 3d, e and Supplementary Fig 5). Triskelions readily fit into this capsid (Fig. 3e), which is amply complemented by the most abundant diameters observed by TEM (Fig. 3b, c).

**Fig. 4:**
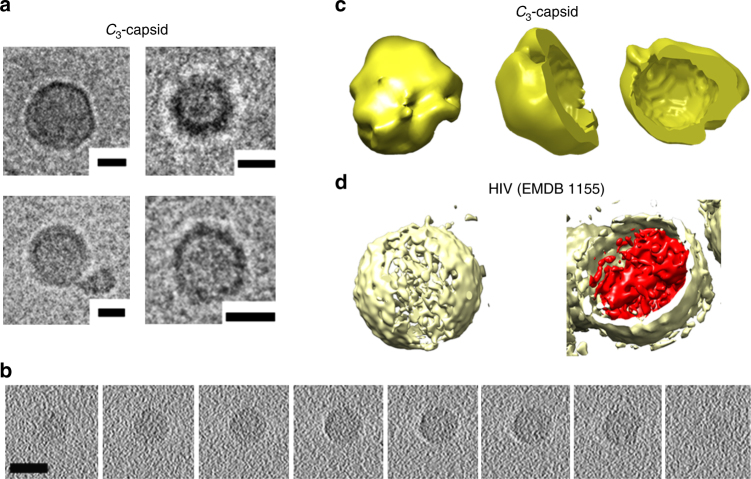
*C*_3_-capsids in 3D. **a** Cryo-electron micrographs of assembled *C*_3_-capsids. Scale bars are 10 nm. **b** Representative cryo-electron tomography (*z*-planes) for an assembled *C*_3_-capsid. Scale bar is 20 nm. **c** 3D rendering of the capsids. Volume reconstruction for a *C*_3_-capsid is fitted to a sphere with a radius of 12 nm showing the exterior of the capsid in yellow and its side and top cross-sections. Molecular graphics and analyses were performed using UCSF Chimera package^39^. Chimera is developed by the Resource for Biocomputing, Visualisation and Informatics at the University of California, San Francisco (supported by NIGMS P41-GM103311). **d** 3D rendering of HIV-1 core shell (EMDataBank entry 1155) shown for comparison. The volume reconstruction, with the exterior and interior shown in yellow and red, respectively. Note: not to scale, HIV particles are >100 nm in diameter

Secondly, regardless of size variations, viral capsids maintain their shell-like morphology. To gain a better insight into this for the design, *C*_3_-capsids were analysed using cryo-TEM and tomography that revealed hollow shells having diameters in the same range observed by high-resolution TEM (Fig. 4a, Supplementary Fig 6). It should be noted that cryo-EM reconstructions provide attainable resolution in the 5–10-nm range^38^, which borders the size of the triskelion shells. Therefore, three-dimensional (3D) volume reconstructions of the obtained images were fitted into 24-nm spheres^39^, matching predominant diameters of 20–24 nm, and were benchmarked against larger (>100 nm) shells of the HIV-1 core assembly used for comparison^40^. Returned reconstructions confirmed the assembly of *C*_3_-capsids as spherical, hollow shells (Fig. 4b–d). In accord with the data, atomic force microscopy (AFM), which provides an ideal probe for the analysis of morphologically defined assemblies, revealed globular capsids with a similarly narrow size distribution under both in-air and in-solution imaging conditions (Supplementary Fig 7a, b). Notably, sizes measured by AFM in solution closely matched those observed by DLS (Supplementary Fig 7b and 4f).

Thus, thirdly, the geometrically conserved shells established for the design manifest a synergy between the assembly and folding of *C*_3_-subunits. Consistent with this, *C*_3_-capsids were found to retain the strong helicity of the elementary *C*_1_-subunit (Supplementary Figs 3a and 8a). Both CD and Fourier transform infra-red spectroscopy (FTIR) spectra for the capsids were characteristic of α-helical conformations, with elements of a β-turn structure that could be assigned in the FTIR spectra being attributable to the dendrimer hub (Supplementary Fig 8b). In particular, the amide I band in the spectra appeared to be red-shifted (1650 ± 2 cm^−1^) from the values expected for a monomeric α-helix (1656 ± 2 cm^−1^), indicating helical adjustments for the coiled-coil formation in the *C*_3_-subunits (Supplementary Fig 8b)^41^. These adjustments proved to be cooperative as reflected in gradual increases in helicity as a function of increasing concentrations of the *C*_1_ (–) strand titrated into the *C*_3_ (+) strand (Supplementary Fig 8c)^42,43^. Plotting these increases versus the concentrations revealed a saturation ratio of 3 for the *C*_1_ (–) to *C*_3_ (+) strands and a single binding affinity value (*K*_D_) of 32 ± 5.6 µM^44^ (Supplementary Fig 8d). At this ratio, the assembly thermally denatured (20–90 °C) returned sigmoidal unfolding curves, with helical spectra recorded every 1 °C sharing the same signal intensity at 202 nm (Supplementary Fig 8e, f), which indicates a clear one-state transition comparable to that observed for the elementary unit (Supplementary Fig 3). Taken together, these findings confirm the cooperative assembly of the *C*_3_-capsids and the complementarity of coiled-coil interactions and cysteine-promoted hydrophobic clustering. More specifically, complementarity ensures that each *C*_3_-subunit is stabilised as a rigid, autonomous structure, while cysteine caps encourage the formation of a hydrophobic network at vertices. Proximity-driven cross-linking of the caps can accompany the clustering, but is not necessary, with the variation of odd and even numbers of cysteines available at the vertices. Indeed, comparative quantifications by Ellman’s test gave up to 25% reductions in the concentration of free thiols in the capsids, confirming a partial disulphide formation. Such a dual stabilisation function—clustering and cross-linking—allows the assembly to maintain the anisotropic packing of spatially restricted *C*_1_-subunits at the vertices facilitating the formation of a well-hydrated shell.

### Antimicrobial activity and mechanism of *C*_3_-capsids

To elicit the biological activity, the design has two main modes of folding, each supporting a specific function. The assembly is stable in solution as well as in the presence of mammalian cell membranes. The capsids, however, are expected to bind to microbial membranes, which is followed by their disassembly into two independent components—the antimicrobial *C*_3_ (+) triskelion that folds and inserts in the microbial phospholipid matrix, and the *C*_1_ (−) strand, the anionic antagonist, which cannot bind to cellular membranes, and instead converts into a random coil remaining in solution. This renders the capsids differential in attacking microbial, but not mammalian, membranes. To probe this behaviour in isolation from intracellular processes^45,46^, which may indirectly involve in the antimicrobial activity, the folding of the components was measured in the presence of zwitterionic and anionic unilamellar vesicles (ZUVs and AUVs) mimicking mammalian and bacterial membranes, respectively. Two lipid types, saturated and unsaturated, were used to assemble the vesicles. 1,2-Dilauroyl-sn-glycero-3-phosphocholine (DLPC) and its 3:1 molar mixtures with 1,2-dilauroyl-sn-glycero-3-phospho-(1′-rac-glycerol) (DLPG) were used for the corresponding assembly of ZUVs and AUVs. Thicker, unsaturated and more asymmetric membranes were assembled from 1-palmitoyl-2-oleoyl-sn-glycero-3-phosphocholine (POPC) and 1-hexadecanoyl-2-(9Z-octadecenoyl)-sn-glycero-3-phospho-(1′-rac-glycerol) (POPG) lipids. All these compositions give fluid-phase membranes at both room and physiological temperatures, allowing for the direct assessment of membrane-disrupting effects in solution. As expected, the *C*_3_ (+) triskelion folded only in AUVs, while the *C*_1_ (−) strand did not fold at all (Supplementary Fig 9). These differences correlated with biological activities. Namely, the *C*_3_ (+) triskelion and the assembled capsids were strongly antimicrobial with no apparent haemolytic activities at micromolar concentrations. Minimum inhibitory concentrations (MICs) were typical of polypeptide antibiotics within the phenotypic tolerance of bacteria to antibiotics^47^, while the *C*_1_ (−) strand was biologically inactive (Table 1).

The seemingly lower MICs of *C*_3_-capsids are for the total peptide concentrations; that is, for 1:3 molar ratios of *C*_3_ (+) triskelions co-assembled with *C*_1_ (−) strands. Therefore, the actual active concentration of the triskelions in the assembled capsids is decreased by to a factor of 4 (Table 1). In principle, higher MICs for the capsids could also be accounted for by a partial antagonistic action of the *C*_1_ (−) strand on the *C*_1_ (+) strand inhibiting membrane binding. To be apparent, however, this action requires substantially higher ratios^25^. Nonetheless, for the reasons of avoiding strong antagonistic effects of the *C*_1_ (−) strand, each heptad of the *C*_1_ (+) strand incorporates an additional lysine residue contributing a positive charge that is not engaged in coiled-coil formation, is solvent exposed and hence is available for binding to microbial membranes (Fig. 2a-d). Since in their native environments antimicrobial peptides are secreted to act within their proteolytic life time (minutes), the antimicrobial action of capsids should be evident within similar timescales. MIC assays cannot access such timescales because these tests are optical density measurements performed over much longer periods of time (24 h).

**Table 1:** Biological activities of peptides used in the study

Cell	Peptide^a^					
	*C*_3_ (+) strand	*C*_3_-capsid	Cecropin B	Daptomycin	Polymyxin B	Gramicidin S
	Minimum inhibitory concentrations, µM^b^					
*P. aeruginosa* (ATCC27853)	3	12	<2	>100	<1	>40
*S. aureus* (ATCC6538)	50	>100	>100	<8	<50	<20
*E. coli* (K12)	1.5	3	<1	>100	<1	<20
*B. subtilis* (ATCC6633)	1.5	3	>50	<8	1.5	>40
*S. enterica* (ATCC700720)	3	3	3	>50	<1	<10
*E. faecalis* (OG1X)	50	>50	<25	>100	>100	ND
*K. pneumoniae* (NCTC 5055)	>12	<25	<1	>50	<1	ND
	(LC_50_), µM^c^					
Human erythrocytes	>250^d^	>250	>250	>250^d^	UD	>20

All tests were done in triplicates

UD undetectable, ND not determined

^a^ *C*_1_ (−) strand was inactive (>250 µM)

^b^Total peptide concentration

^c^Median (50%) cell death compared to untreated cells

^d^Haemolysis of <10% observed at higher concentrations

To gain a better insight into killing kinetics at the cellular level, we monitored the lysis of Gram-negative *Escherichia coli* and Gram-positive *Bacillus subtilis* using one-stain (propidium iodide) and two-stain (LIVE/DEAD® BacLight^TM^) bacterial viability assays in conjunction with fluorescence microscopy (Supplementary Fig 10). For both bacteria, the capsids used at the MIC (3 µm) caused progressive lysis of up to 80% of the total number of cells within the first 20 min reaching nearly complete killing rates in 30 min (Supplementary Fig 10). Collectively, the results of the biological tests indicate strong and rapid antimicrobial responses of the *C*_3_-capsids, suggesting a physical, membrane-mediated mechanism of action. Indeed, once in membranes *C*_1_ (+) strands are meant to refold and insert in phospholipid bilayers in a transmembrane manner^46^. Because all three legs in the *C*_3_ (+) triskelion are identical, each has the same ability to bind and insert. Because the length of each strand matches the thickness of the phospholipid bilayers (3–4 nm), this ability is not restricted, but facilitated by the other two strands of the triskelion that remain lipid bound. Finally, given that in membranes all the strands would have the same orientational preference, for which they compete, triskelions are anticipated to inflict an immediate membrane damage leading to the progressive removal of the bilayer. Consequently, *C*_3_-capsids are predicted to porate phospholipid bilayers with the formation of large and expanding transmembrane pores.

**Fig. 5:**
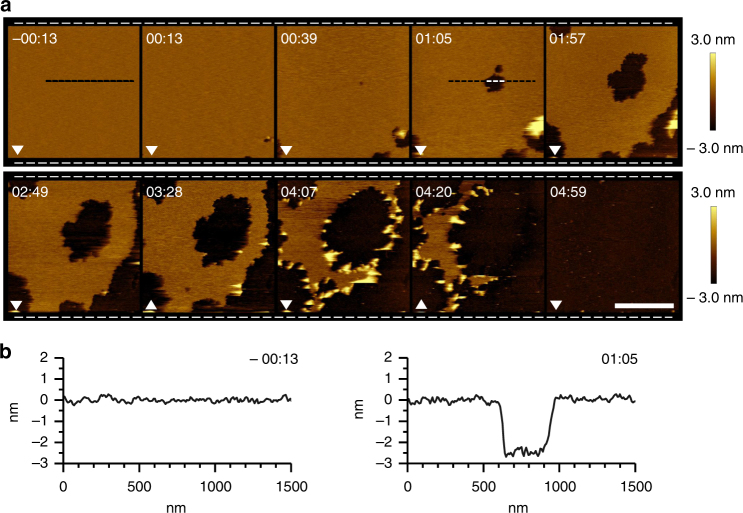
*C*_3_-capsids porating phospholipid bilayers. **a** In-water AFM imaging of supported lipid bilayers treated with *C*_3_-capsids (3 µM total peptide). Topography (height) images, captured at 13 s per frame, are shown. The time stamp corresponds to the middle line of each AFM scan, referenced to the time (00:00) of capsid injection. White arrowheads indicate the AFM scan direction. The scale bar is 1 μm. **b** Cross-sections along the lines marked in (**a**, −00:13 and 01:57) before (left) and after (right) the addition of capsids

It should be noted, however, that microbial membranes are curved 3D structures that do not exceed 2 µm in size. Poration in these membranes can lead to rapid membrane rupture and collapse before individual pores can expand visibly. Longer time and length scale studies are necessary to observe the predicted effect, which can be accessed in supported lipid bilayers (SLBs). These preparations give ideal models for fluid-phase membranes and are flat (to within ~0.1 nm) in their unperturbed state, thereby ensuring the most accurate depth measurements of forming pores in real time by AFM^45^. With this in mind, we visualised the effect of *C*_3_-capsids on SLBs prepared by the surface deposition of AUVs in aqueous solution^46^, using an AFM adapted for high-speed scanning^48^ and compiling videos (Supplementary Movie 1) based on sequences of rapidly acquired AFM images^49^. Upon the addition of the capsids, poration was apparent within seconds of incubation, indicating the instantaneous disassembly of the capsids on the membranes (Fig. 5a and Supplementary Movie 1). The formed pores grew in size and merged resulting in the complete removal of the available phospholipid matrix under 5 min (Fig. 5a). The depths of the pores were consistently ~3 nm, indicating that *C*_1_ (+) strands spanned the bilayer (Fig. 5b and Supplementary Movie 1).

The data prompt a mechanism according to which *C*_3_-capsids at once disassemble upon landing on membranes, whereupon *C*_3_-triskelions re-assemble into transmembrane pores—all within several minutes. The orientation of *C*_1_ (+) strands incorporated into the bilayer is likely to be in a dynamic equilibrium with the other strands in the same *C*_3_ (+) triskelion whose membrane binding must be equally satisfied. On the one hand, such a demand for each *C*_1_ (+) strand to bind and fold with the lipids promotes profound membrane disruption manifesting in rapidly expanding pores (Fig. 5). On the other hand, a thicker peptidoglycan layer on the surface of Gram-positive bacteria that is rich in anionic teichoic polymers might initiate the premature disassembly of the capsids, while inhibiting the migration of folded triskelions to the cytoplasmic membrane^50^. Partly, this may explain the less pronounced activities of the capsids against Gram-positive *Staphylococcus aureus* and *Enterococcus faecalis* (Table 1). By contrast, Gram-positive *B. subtilis*, whose peptidoglycan undergoes continuous transformations from thick to thin layers^51^, was noticeably more susceptible to the treatment of the capsids (Table 1). Encouragingly, this property of the capsids to respond differentially to bacterial membranes can be harnessed for targeting Gram-negative membranes, in which case membrane-selective capsids could be developed.

## Discussion

The demonstrated concept offers a molecular platform for engineering bespoke antimicrobial capsids that can be assembled from relatively short amino-acid sequences. Given that there are thousands of antimicrobial peptides reported in the literature^5,6^, each of these may be used to construct a self-assembling capsid protomer. Antimicrobial sequences are certainly not limited to stand-alone peptides, naturally occurring or designed, and can be sourced from native proteins exhibiting complementary properties. For example, short peptide fragments of transmembrane proteins or bacteriostatic proteins from biological fluids can be redesigned to assemble into biologically functional nanoscale shells^17,52^. The use of antagonistic sequences that co-fold with antimicrobial sequences introduces another level of control when compared to structures assembled exclusively from antimicrobial components^17^. Resulting hetero-oligomers give rise to discrete protomers for which number in a final assembly can be pre-defined in a manner similar to the aggregation number of capsids used to describe viruses^9^. Not only this supports geometrically conserved structures with a narrow size distribution, as demonstrated here, but can also provide a means of tuning discrete capsids or their ensembles within close *T* > 1 ranges.

In principle, this ability would be important in addressing broader aspects of antimicrobial activity including the targeting of intracellular pathogens. Conventional antibiotics are of little help against these, and particularly against dormant forms that are phenotypically tolerant to antibiotic treatments^53^. Similar to antimicrobial peptides, which, unlike antibiotics, attack both growing and mature bacteria^29^, the designed peptide capsids may prove effective in this regard: their differential membrane binding is directly related to their ability to traverse mammalian membranes and reach the cytoplasm without cytotoxicity. Antimicrobial sequences have built-in cell-penetrating properties, so do the designed capsids. Furthermore, the capsids may encapsulate genetic cargo or a reporter and deliver it into the cytoplasm of human cells, which may provide a dual function of cell penetration and delivery. To probe the feasibility of intracellular delivery, the capsids were co-assembled with a model RNA that was fluorescently labelled. The resulting capsids promoted an active RNA delivery into HeLa cells within hours (Supplementary Fig 11a). The fluorescent RNA appeared as particulate cytoplasmic spreads in the cytoplasm suggesting an endocytic uptake^14^. Flow cytometry measurements showed that the transfection efficiency of the capsids was at least comparable with that of commercial transfection reagents (Supplementary Fig 11b, c), whereas RNA mixed with the *C*_1_ (−) strand, as a negative control, showed no transfection (Supplementary Fig 11d). Thus, the capsids are effectively infectious agents whose cellular and intracellular targeting can be enhanced by incorporating specialist moieties allowing for receptor-mediated delivery.

In more practical terms, whereas better antibiotics inevitably lead to a fitter resistance, the concept entails a promising discovery paradigm. The clinical potential of antimicrobial capsids owes to the lack of widespread microbial resistance to antimicrobial sequences^5^. Membrane disrupters impose a prohibitive cost for bacterial resistance to develop^6^, but yet their action is bound to the same “S”-to-“I” transition of individual peptide monomers. By contrast, pre-concentrating the monomers into capsids introduces a principally more challenging scenario of virtually built-in pores that can instantaneously convert into irreparable membrane lesions in bacterial cells.

Indeed, the designed capsids are weakly cationic, which might allow them to cross biological barriers more effectively (Supplementary Table 1). For example, particles with strong cationic surfaces are not effective in crossing blood–brain barrier, which remains a major obstacle for their therapeutic uses^14^. Native viruses have isoelectric points (pI) below 8, but which can extend due to sorption processes, while for highly infectious viruses such as polioviruses pI can be >8^54^. The designed capsids possess a weak positive charge that is sufficient to bind to anionic bacterial surfaces, and upon binding the capsids disassemble with a rapid increase in pI (to 11 as that of the *C*_3_ (+) strand), thus effectively binding membrane phospholipids and disrupting the bilayer.

Most peptide-based antibiotics destroy bacterial cell membranes (colistin) or inhibit their construction (vancomycin). With the increasing usage of membrane-active antibiotics in clinic (daptomycin, polymyxins) and a stronger drive towards antibiotics effective against Gram-negative bacteria, there is a pressing need to extend search strategies to alternative approaches involving macromolecules that are different from antibiotics altogether and may build upon similar, membrane-disrupting mechanisms. For all these reasons, the described mechanistic design lends itself to emerging classes of antimicrobial agents and materials with a rich repertoire of exploitable properties.

## Methods

### Peptide synthesis

All the peptides were assembled on a Liberty microwave peptide synthesiser (CEM Corp.) using standard Fmoc/tBu solid-phase protocols with HBTU/DIPEA as coupling reagents (Supplementary Fig 1). Rink amide 4-methylbenzhydrylamine resin was used throughout. Standard Allyl-based orthogonal protocols were employed for the synthesis of the *C*_3_-triskelion on resin, with Fmoc-Lys(Alloc)-OH used to generate the *C*_3_-dendrimer hub (Supplementary Fig 1). The hub and the *C*_1_ (−) strand were acetylated using a solution containing acetic anhydride (5%, v/v) and pyridine (5%, v/v) in DMF. Upon cleavage and deprotection of cysteine-free peptides (95% TFA, 2.5% TIS, 2.5% water) and the *C*_1_ (−) strand (95% TFA, 2.5% TIS, 2.5% EDT), the peptide identities were confirmed by analytical RP-HPLC and MALDI-ToF (Supplementary Fig 2).

MS [M+H]^+^: *C*_1_ (−) strand—*m*/*z* 2545.8 (calc.), 2546.1 (observed); Cys-free *C*_1_ (−) strand—*m*/*z* 2371.6 (calc.), 2372.5 (observed); *C*_1_ (+) strand—*m*/*z* 2447.1 (calc.), 2447.3 (observed); *C*_3_-triskelion—*m*/*z* 8302.6 (calc.), 8302.6 (observed). MS [M+Na]^+^: *C*_1_ (−) strand—*m*/*z* 2568.8 (calc.), 2569.3 (observed); Cys-free *C*_1_ (−) strand—*m*/*z* 2394.6 (calc.), 2394.6 (observed); *C*_3_-triskelion—*m/z* 8325.5 (calc.), 8324.9 (observed).

### High-performance liquid chromatography

Analytical and semi-preparative gradient RP-HPLC was performed on a JASCO HPLC system using Vydac C18 analytical (5 μm) and semi-preparative (5 μm) columns. Both analytical and semi-preparative runs used a 10–60% B gradient over 40 min at 1 and 4.7 mL min^–1^, respectively, with detection at 230 and 220 nm. Buffer A—5% and buffer B—95% aqueous CH_3_CN, 0.1% TFA.

### CD spectroscopy

CD spectra were recorded on a Chirascan Plus spectropolarimeter (Applied Photophysics Ltd.) equipped with a Peltier temperature controller. All measurements were taken in millidegrees using 1 nm step, 1 nm bandwidth, 1 s time/point and four acquisitions. All measurements were recorded in a quartz cuvette with 0.05 cm pathlength. Aqueous peptide solutions (300 µL, 100 µM total peptide, unless stated otherwise) were prepared in filtered (0.22 µm) 10 mM 3-(N-morpholino)propanesulfonic acid (MOPS), pH 7.4, room temperature. After baseline subtraction, the spectra were converted to mean residue ellipticities (MRE, deg cm^2^ dmol^−1^ res^−1^) by normalising for the concentration of peptide bonds and the cell pathlength. CD spectra at variable temperature were recorded with a 1-°C step from 20 to 90 °C with 180 s equilibration time for each spectrum. Thermal denaturation curves were obtained by plotting the MRE at 222 nm as a function of temperature, the first derivative of which provided *T*_M_ values. Binding titrations were performed using module B end-station spectrophotometer at B23 beamline (Diamond Light Source, UK) and a cylindrical cuvette with a 0.01-cm path. All measurements were performed by titrating the *C*_1_ (−) strand into the *C*_3_-triskelion (25 µM). Each sample for a specific molar ratio was incubated for 15 min prior to measurement. All measurements were taken in ellipticities and *K*_D_ values were calculated using Hills equation^44^. The thermal denaturation curve was fitted to standard two-state denaturation equations (dose response and Hills equations). The calculations returned a nearly sigmoidal curve (Supplementary Fig 3)^30^. The coefficient of determination (*R* ^2^) used as the measure of goodness of fit was 0.999 for the data versus 1 for an ideal fit. CD spectra in the presence of ZUVs and AUVs are for lipid–peptide (L/P) molar ratios of 100.

### FTIR spectroscopy

All FTIR spectra were collected using a Tensor-37 series FTIR spectrophotometer with a BioATR II unit (Bruker Optics, UK) as the sampling platform with a photovoltaic mercury cadmium telluride (MCT) detector and a Bruker Optics workstation, which was equipped with OPUS software. Low volume (20 μL) peptide samples (100 μM) were placed in a circular sampling area of radius 2 mm with a pathlength of 6 μm. The temperature of the sample was maintained at 20 °C by means of flow connectors to a circulating water bath. All FTIR spectra were collected between 4000 and 850 cm^−1^ with a resolution of 4 cm^−1^, scanner velocity 20 kHz, 128 scans, phase resolution 32 and zero filling factor 4.

### Lipid vesicle preparation

The lipids DLPC and DLPG, and POPC and POPG used for liposome construction were from Avanti Polar Lipids. The lipids were weighted up, dissolved in chloroform–methanol (2:1, v/v), dried under a nitrogen stream and placed under vacuum overnight. The resulting film was hydrated to 10 mg mL^–1^ total lipid concentration in 10 mM phosphate buffer, pH 7.4. The suspension was then extensively vortexed, sonicated (30 °C) and extruded (15 times) through polycarbonate filters (0.05 μm) using a hand-held extruder (Avanti Polar Lipids) to give a clear solution containing small unilamellar vesicles (SUV), which were analysed (50 nm) by photon correlation spectroscopy using a Zetasizer Nano (ZEN3600, Malvern Instruments, UK) following the re-suspension of vesicles to a final concentration of 1 mg mL^−1^. DLS batch measurements were carried out in a low-volume disposable cuvette at 25 °C. Hydrodynamic radii were obtained through the fitting of autocorrelation data using the manufacture’s software, Dispersion Technology Software (DTS version 5.10).

### Determination of free thiols

A stock solution of 5,5′-dithio-bis(2-nitrobenzoic acid) (Ellman’s reagent from Pierce) was prepared to a final concentration of 2 mM in LC-MS grade water with 50 mM sodium acetate. Fifty microlitres of the solution was mixed with 10 mM MOPS (pH 7.4, 100 µL) and LC-MS grade water (840 µL), to take background absorbance. Pre-assembled capsids (10 µL, 100 µM, without TCEP) were then added to the solution (final volume of 1 mL); 200 µL of the final solution was scanned in the 280–600 nm region (automatic background subtraction) with recording absorbance at 412 nm. The absorbance for each sample was calculated and the results were averaged and divided by 14,150 M^−1^ cm^−1^ (extinction coefficient of 2-nitro-5-thiobenzoate) and 1 (number of thiols in peptide) to obtain the final concentrations. Free thiol was quantified using a cysteine standard curve prepared according to the proprietary protocol.

### High-resolution TEM

Electron micrographs were recorded using a FEI Tecnai 20 twin lens scanning transmission electron microscope, operated at 200 kV. Droplets of solution (8 µL) were placed on glow discharged Cu grids coated with pioloform and carbon film. After 1 min, the excess solvent was removed using a blotting paper and the grid was stained with uranyl acetate (1%, wt) for 15 s before blotting the excess stain. High-resolution images were recorded using a proprietary FEI Eagle 4k × 4k CCD.

### Cryo-TEM

Cryo-TEM samples were prepared by plunge freezing performed using a VITROBOT mark IV (FEI Company)—into liquid N_2_-cooled liquid ethane. Droplets (5 µL) of peptide solutions (100 µM) were placed on glow discharged lacey carbon grids and left for 2 s before blotting (2 s) and plunging. This yielded samples in which peptide capsids are embedded in the vitreous ice suspended inside the holes of the carbon. The sample grid was then transferred (without warming) into a Gatan 626 cryo-holder and visualised at 200 kV in a Tecnai T20 (FEI company) transmission electron microscope fitted with an Eagle 4k × 4k camera (FEI).

### Cryo-TEM tomography

The samples were prepared as above and tilt series were collected automatically at 3° increments over an angular range of −42° to 55°. The electron dose was 2.75 electrons per square Ångstrom per second per image. Projection alignment, reconstruction and image processing were done using IMOD. Visualisation, surface reconstruction and final segmentation were performed in Amira 3.0 (TGS, San Diego, CA).

### In-air AFM

AFM images were obtained for a drop of peptide solution (8 µL, 100 µM) placed on a clean silicon wafer with the buffer excess removed by blotting. All measurements were carried out on a Cypher instrument (Asylum research) in a tapping mode using super-sharp PPP-NCHR type cantilevers (Nanosensors™: resonant frequency ~330 kHz, tip radius of curvature <5 nm, force constant 42 N m^−1^). Images were flattened via line-by-line subtraction of first-order fits to the background, using SPIP software, version 6.0.2.

### Preparation of SLBs for AFM in water

SLBs were formed on mica as described elsewhere^45^ from a vesicle solution of 4 mg mL^−1^ DLPC/DLPG (3:1, molar ratio) and for POPC/POPG (3:1, molar ratio) SLBs. The vesicle solution was incubated on cleaved mica for 30 min in 10 mM MOPS pH 7.2, with 5 mM MgCl_2_ and 5 mM CaCl_2_. After absorption, the solution was washed 10 times with 10 mM MOPS pH 7.2, with 150 mM NaCl, to remove unfused vesicles from the solution. Mica discs (Agar Scientific, Stansted, UK) were glued to a metal puck, and freshly cleaved prior to lipid deposition.

### In-water AFM on SLBs

Topographic images of SLBs in water were recorded on a Multimode 8 AFM (Bruker AXS, CA, USA) with a modified AFM head designed for the photothermal actuation of miniaturised cantilevers^46^. Bruker FASTSCAN-D probes (0.25 N/m) (Bruker AFM probes, CA, USA) were photothermally driven for Tapping mode at ~90 kHz with a typical amplitude setpoint of ~300 mV. Capsids were introduced into the sample volume via a channel in the custom fluid cell and diluted in the existing buffer solution (150 mM NaCl, 10 mM MOPS pH 7.2) to the final concentration stated. A negative Tapping mode setpoint was applied to raise the cantilever above the sample surface during capsid injection. Images of 2 μm size (256 × 256 pixels) were captured in alternate scan directions at a line rate of 19.3 Hz. Images were processed using Gwyddion (http://gwyddion.net/) for zeroth-order flattening to centre data, and first- or second-order plane fitting to remove tilt or bow. The time-lapse AFM video was generated from the image sequence by using Fiji—Image J^47^. Cross-section measurements were carried out using NanoScope Analysis or Gwyddion (http://gwyddion.net/) and plotted using Origin (OriginLab, MA, USA).

### MIC assay

MICs were determined by broth microdilution on different bacteria according to the Clinical and Laboratory Standards Institute. Typically, 100 μL of 0.5–1 × 10^6^ CFU mL^–1^ of each bacterium in Mueller Hinton media broth (Oxoid) were incubated in 96-well microtitre plates with 100 μL of serial two-fold dilutions of the peptides (from 100 to 0 μM) at 37 °C on a 3D orbital shaker. The absorbance was measured after peptide addition at 600 nm using a Victor 2 plate reader (Perkin-Elmer). MICs were defined as the lowest peptide concentration after 24 h at 37 °C. All tests were done in triplicate and the results are summarised in Table 1.

### Haemolysis assay

Haemolysis was determined by incubating a 10% (vol/vol) suspension of human erythrocytes with peptides. Erythrocytes were rinsed four times in 10 mM phosphate buffer saline (PBS), pH 7.2, by repeated centrifugation and re-suspension (3 min at 3000×*g*). Erythrocytes were incubated at room temperature for 1 h in either deionised water (fully haemolysed control), PBS, or with peptide in PBS. After centrifugation at 10,000×*g* for 5 min, the supernatant was separated from the pellet, and the absorbance was measured at 550 nm. Absorbance of the suspension treated with deionised water defined complete haemolysis. The values given in Table 1 correspond to concentrations needed to kill a half of the sample population (50% lysis of human erythrocytes) and are expressed as median lethal concentrations—LC_50_. All tests were done in triplicate and the results are summarised in Table 1.

### Fluorescence imaging of bacteria

Bacterial cultures were centrifuged to give a cell pellet, which was washed twice with 10 mM phosphate buffer (pH 7.4) before being reconstituted in the buffer. One-hundred microlitres of the solution was dispensed in an 8-well chamber (LabTek) with diluted (1/500) propidium iodide (PI) (1 mg mL^–1^, Invitrogen CA, USA). The chambers with surface-settled bacteria were mounted on a confocal microscope (IX 81, Olympus) equipped with an incubation chamber at 37 °C. PI fluorescence emission was monitored at 625 nm for 60 min after the addition of the peptide to a final concentration of 10 µM. Recorded images were processed by ImageJ (NIH, USA, http://imagej.nih.gov/ij/) to plot the number of fluorescent (stain-dead) cells as a function of time. The values are expressed as a percentage of the total number of cells (taken as 100% for each point).

### Bacterial viability LIVE/DEAD® BacLight™ assays

Bacterial cultures (1 mL) were centrifuged to give a cell pellet, which was washed twice with 10 mM phosphate buffer (pH 7.4) before being reconstituted in the same buffer to give OD600 nm = 0.008. A 100-µL aliquot of the solution was dispensed in an 8-well glass chamber (LabTek) with LIVE/DEAD® BacLight™ bacterial viability kit (Invitrogen). The chambers with surface-settled bacteria (20 min) were mounted on a confocal microscope (Olympus) equipped with an incubation chamber at 37 °C. SYTO®9 and the fluorescence emission of PI were monitored at 515 and 625 nm, respectively, at different time points for 50 min after the addition of the capsids. Recorded images (XY) were analysed using Fiji software to plot the number of dead cells as a percentage of the total number of cells (taken as 100% for each point) after subtracting the total number of dead cells in buffer alone (with no capsids added).

### Flow cytometry

Quantification of labelled siRNA uptake was performed by flow cytometry. AF647-siRNA was assembled with the capsids by immediately adding to the buffered capsid components at a peptide/nucleic acid (P/N) charge (+) ratio of 8:1 as previously described^14^. For a negative control, AF647-siRNA was incubated with the *C*_1_ (−) strand at the same charge (−) ratio. For a positive control, AF647-siRNA was assembled with Lipofectamine® RNAiMax (Lipo) according to the proprietary protocols. After 4-h incubations with the obtained preparations, cells were trypsinised and re-suspended in 200 µL PBS. AF647 fluorescence was measured by flow cytometry on a Cytek DxP8 FACScan (USA) and analysed using FlowJo software (USA). At least 10^5^ events were gated from each ≥10^6^ subset measured for each sample by forward scatter and side scatter on the 488 or 633 nm lasers to extract and analyse 10^4^ single viable cells. From the untreated control cells, incubated with 200 µL Opti-MEM containing no siRNA and no peptide, a negative population was taken as 0% negative. This gate was then used on all other samples to measure the relative population of negative (no siRNA) and positive (siRNA) cells, which was expressed in percentage.

### Data availability

The authors declare that the data supporting the findings of this study are available within this paper and its supplementary information files as well as from the authors.

## Electronic supplementary material

Supplementary Information

Description of Additional Supplementary Files

Supplementary Movie 1
