# Supplementary material for: Antimicrobial peptide capsids of de novo design
Source: Nat Commun. 2017 Dec 22;8:2263. doi: 10.1038/s41467-017-02475-3 (PMC5741663; doi:10.1038/s41467-017-02475-3)
Supplement: Supplementary file 1 — Supplementary Information [file 41467_2017_2475_MOESM1_ESM.pdf]

Supplementary material

Antimicrobial peptide capsids of *de novo* design

De Santis et al.

Supplementary Table 1 ζ-potential for constructs used in this study

Construct	ζ-potential, mV
C_3 (+) strand	22.9 ± 1.4
C_1 (-) strand	-24.1 ± 1.1
C_3-subunit	1.3 ± 0.2

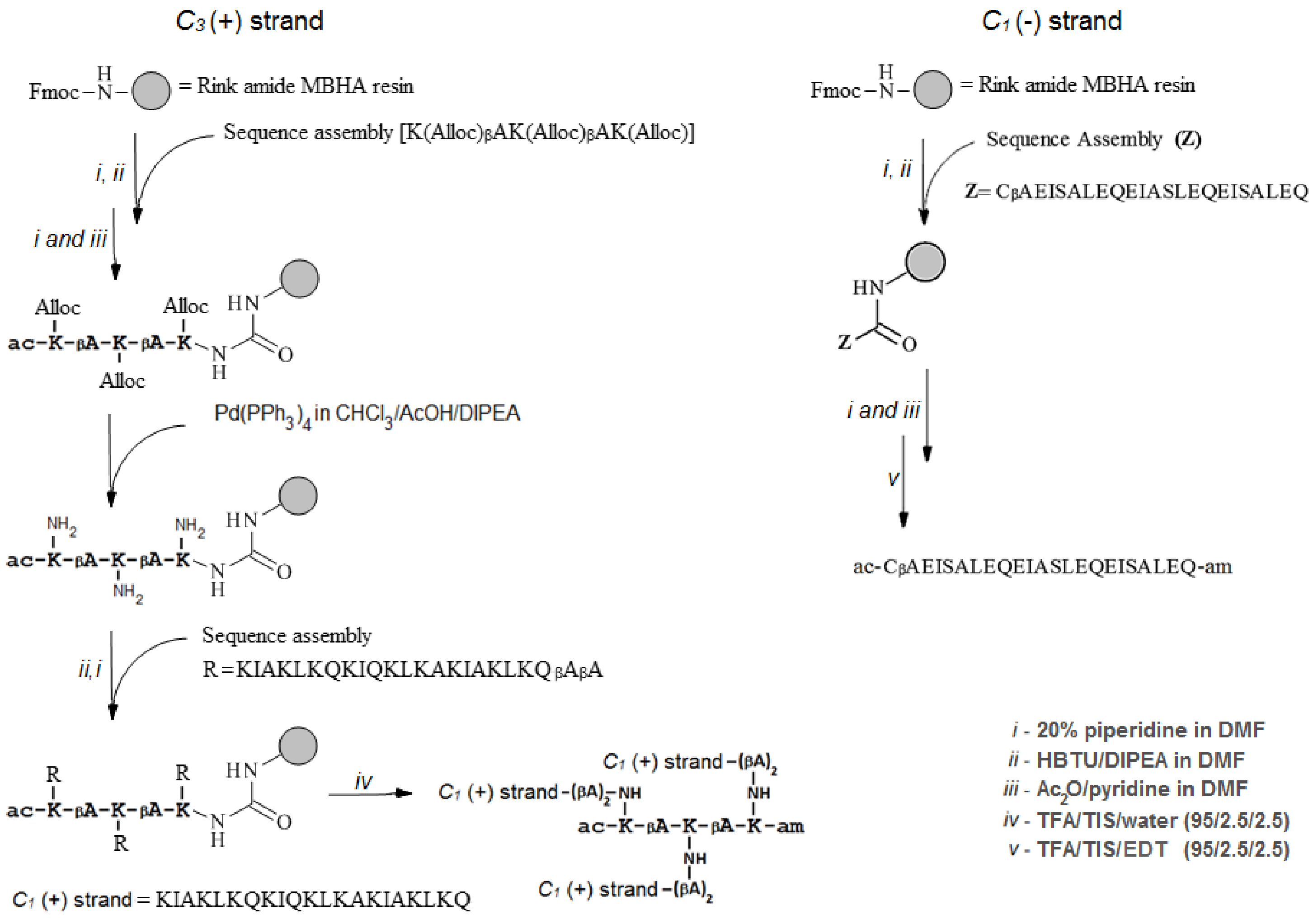

Supplementary Figure 1. Chemical synthesis of capsid-assembling molecules. Synthesis schematics for C_3 (+) strand and C_1 (-) strand.

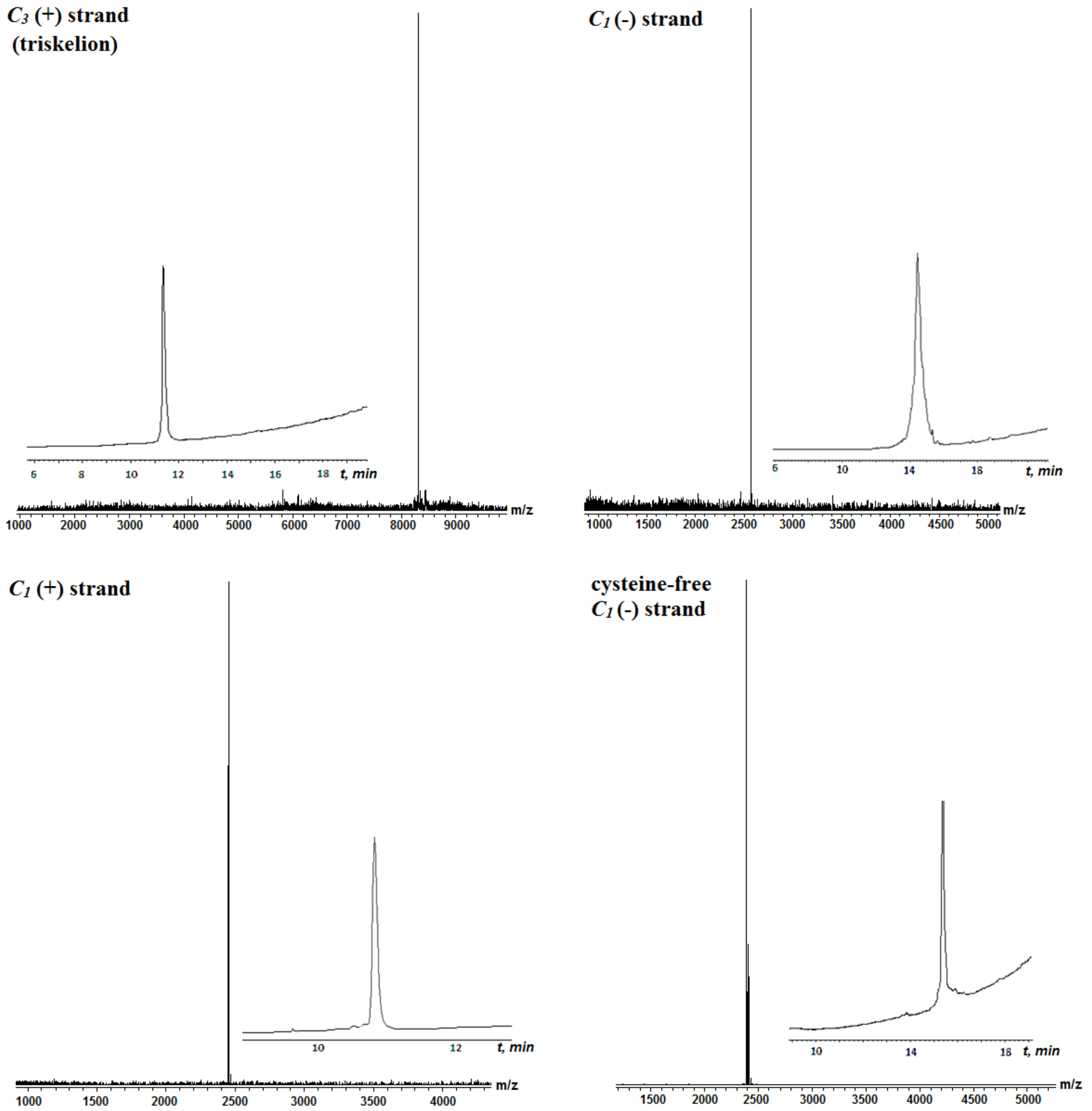

Supplementary Figure 2. Peptide characterisation. RP-HPLC traces and MALDI-ToF spectra for the purified peptides used in the study.

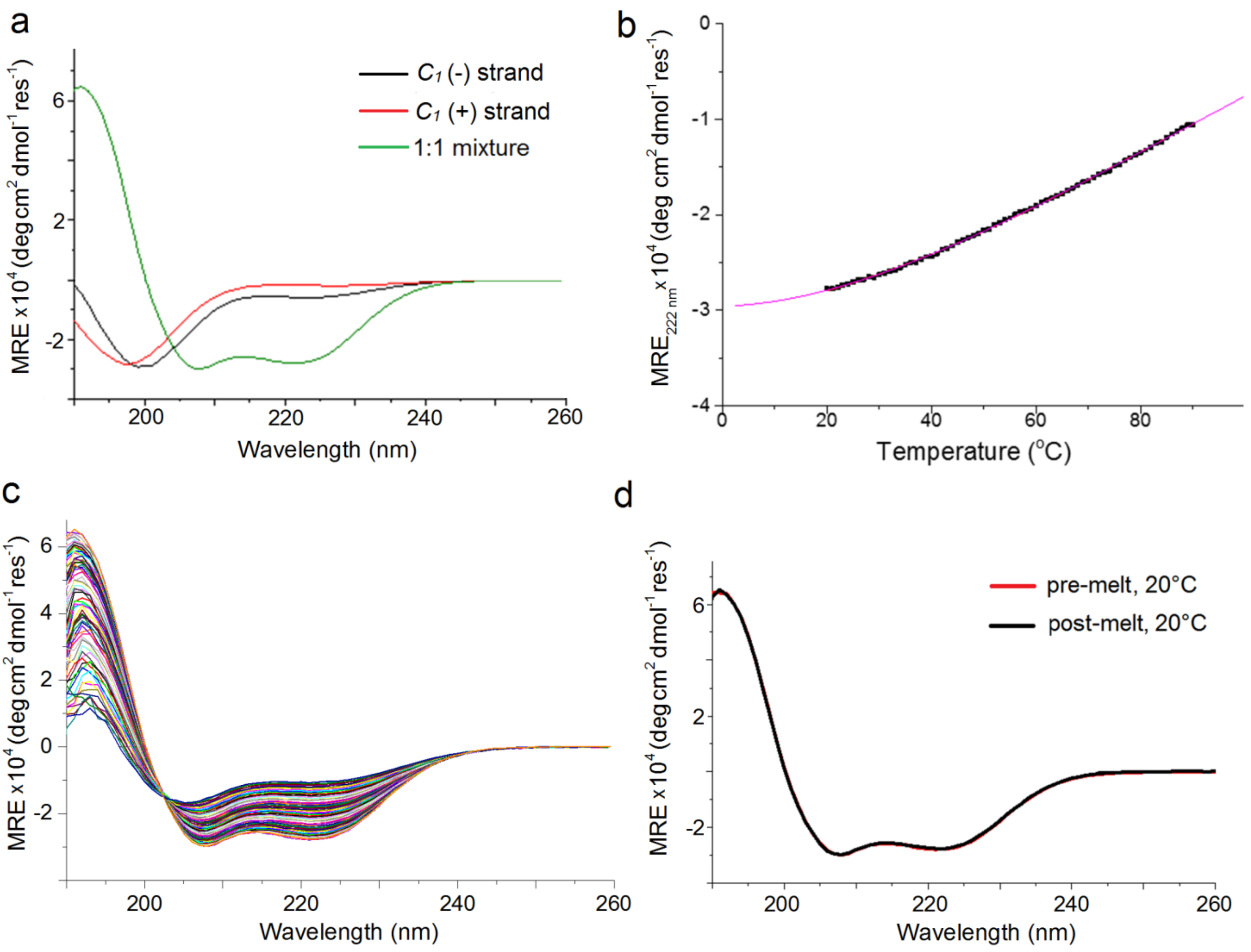

Supplementary Figure 3. Folding of elementary capsid subunits. **a** CD spectra for the C_1 (-) strand (black line), C_1 (+) strand (red line) and their equimolar mixture (green). **b** Thermal unfolding curve for the mixture (the coiled coil subunit) as a function of temperature at 222 nm. Fitting the data to standard two-state denaturation equations returned a nearly sigmoidal curve (magenta) (*30*). The coefficient of determination (R^2) used as the measure of goodness of fit was 0.999 for the data versus 1 for an ideal fit. **c** CD spectra following the thermal unfolding with individual spectra recorded every 1°C. Note the isodichroic point at 202 nm. **d** CD spectra recorded for the mixture before (red) and after (black) the melt. Key: 100 µM peptide (total) in 10 mM MOPS, pH 7.4; with 1 mM TCEP used for C_1 (-) strand and its mixtures with C_1 (+) strand.

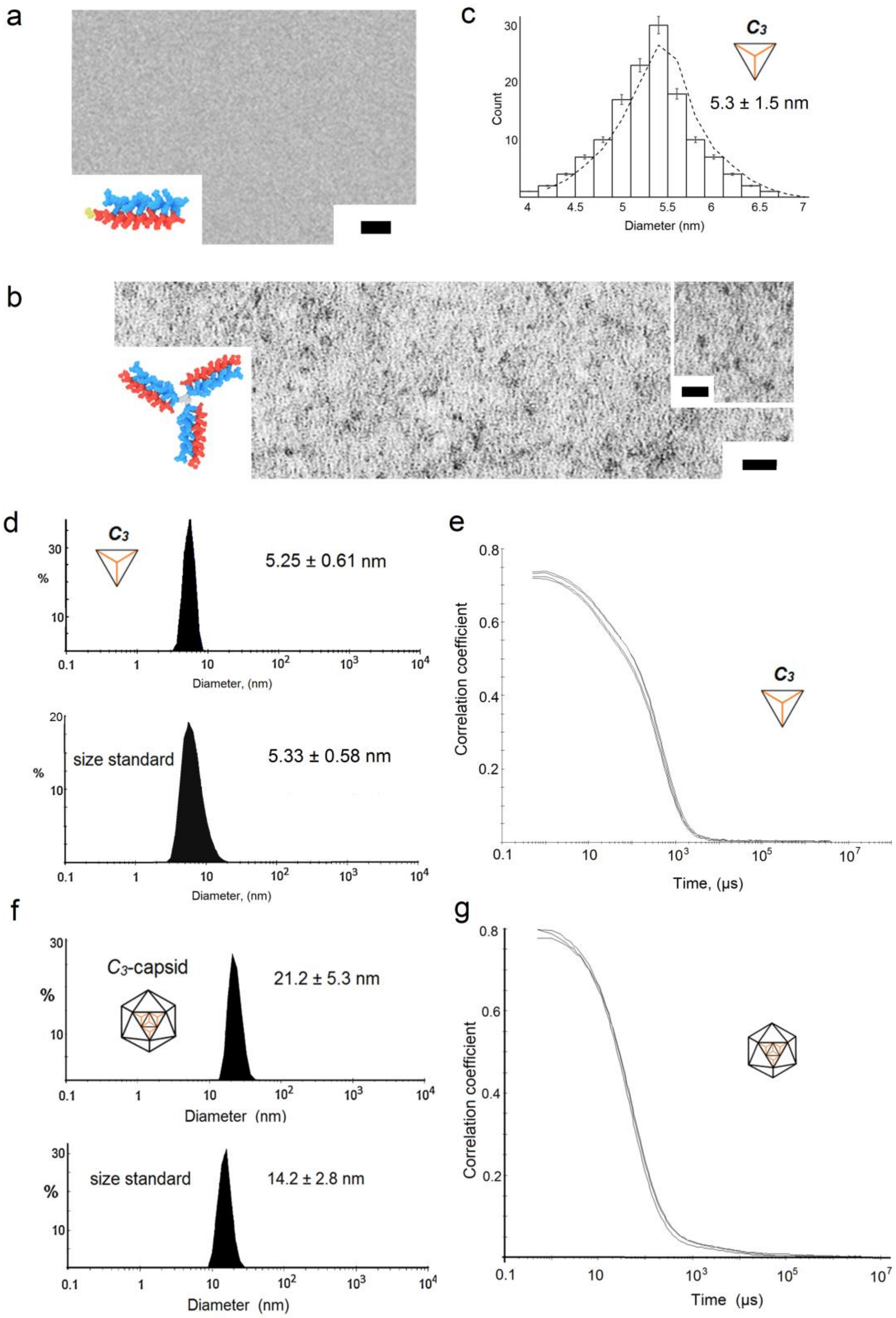

Supplementary Figure 4. Assembly of elementary subunits and capsids. **a** Electron micrographs for C_1-subunit assembled from C_1 (-) strand and C_1 (+) strand. Scale bar is 50 nm. **b** Electron micrographs for C_3-subunit assembled from C_3 (+) strand (triskelikon) and cysteine-free C_1 (-) strand. Scale bars: 20 nm and 10 nm for the inset. **c** Size distributions of cysteine-free C_3-subunit determined by TEM. Each bar represents the mean ± standard error of at least three independent measurements, each done in triplicate. **d** Size distributions determined by DLS for the C_3-subunit and bovine serum albumin used as a size standard (2 mg mL^{-1}) in 10 mM phosphate buffer (pH 7.4). **e** DLS correlograms showing high intercepts for the C_3-subunit. **f** Size distributions determined by DLS for assembled C_3-capsids and a monoclonal IgG (0.5 mg mL^{-1}) in 10 mM phosphate buffer (pH 7.4). The antibody with the known diameter of 14.2 nm was used as a size standard for comparison (Malvern Instruments Ltd). **g** DLS correlograms showing high intercepts for C_3-capsids. Key: 100 μM peptide (total) in 10 mM MOPS, pH 7.4; with 1 mM TCEP used for C_1 (-) strand and its mixtures with C_1 (+) strand.

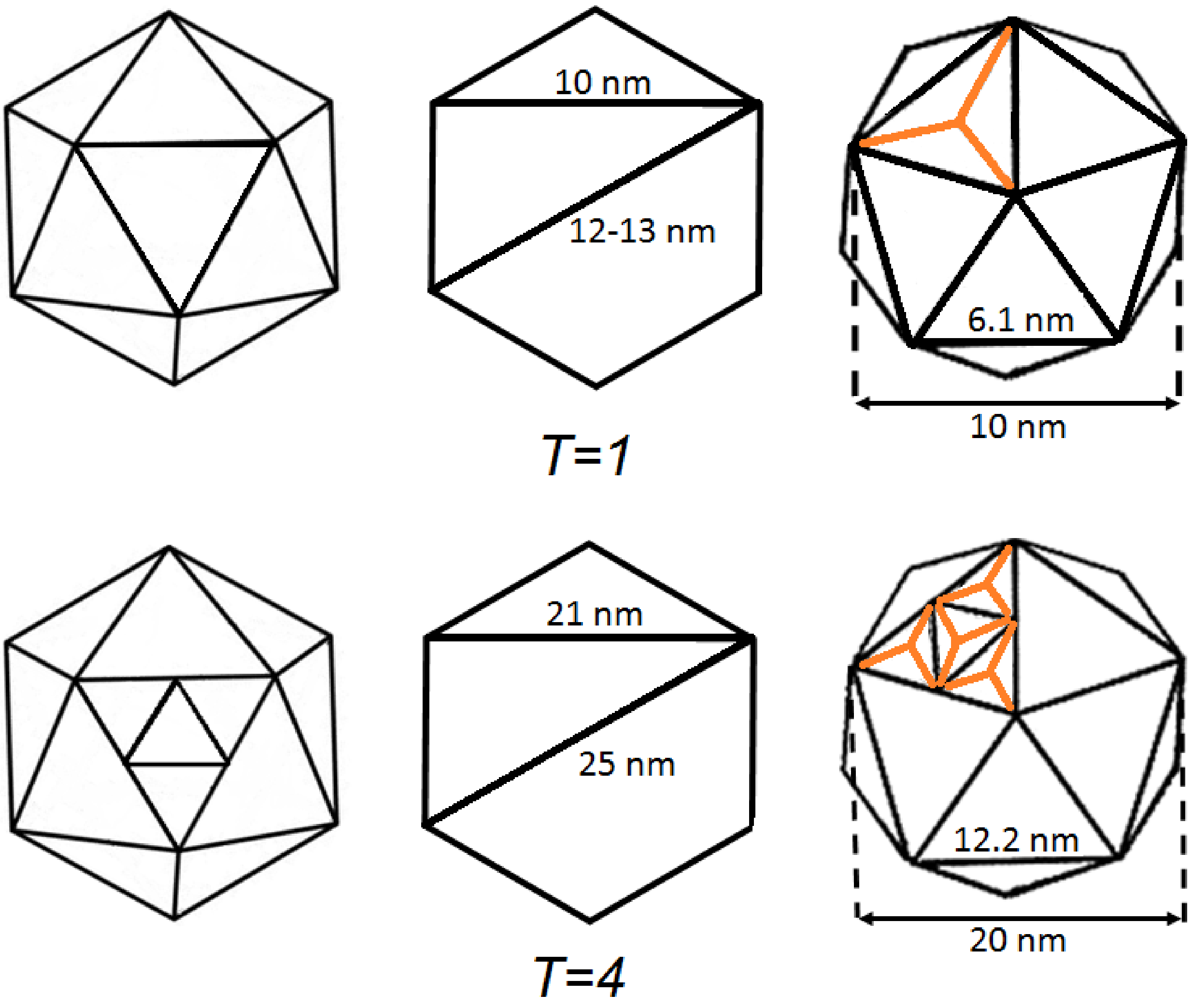

Supplementary Figure 5. Capsid construction parameters. Schematics of *T=1* and *T=4* icosahedral capsids with size dimensions calculated from the C_3-subunit.

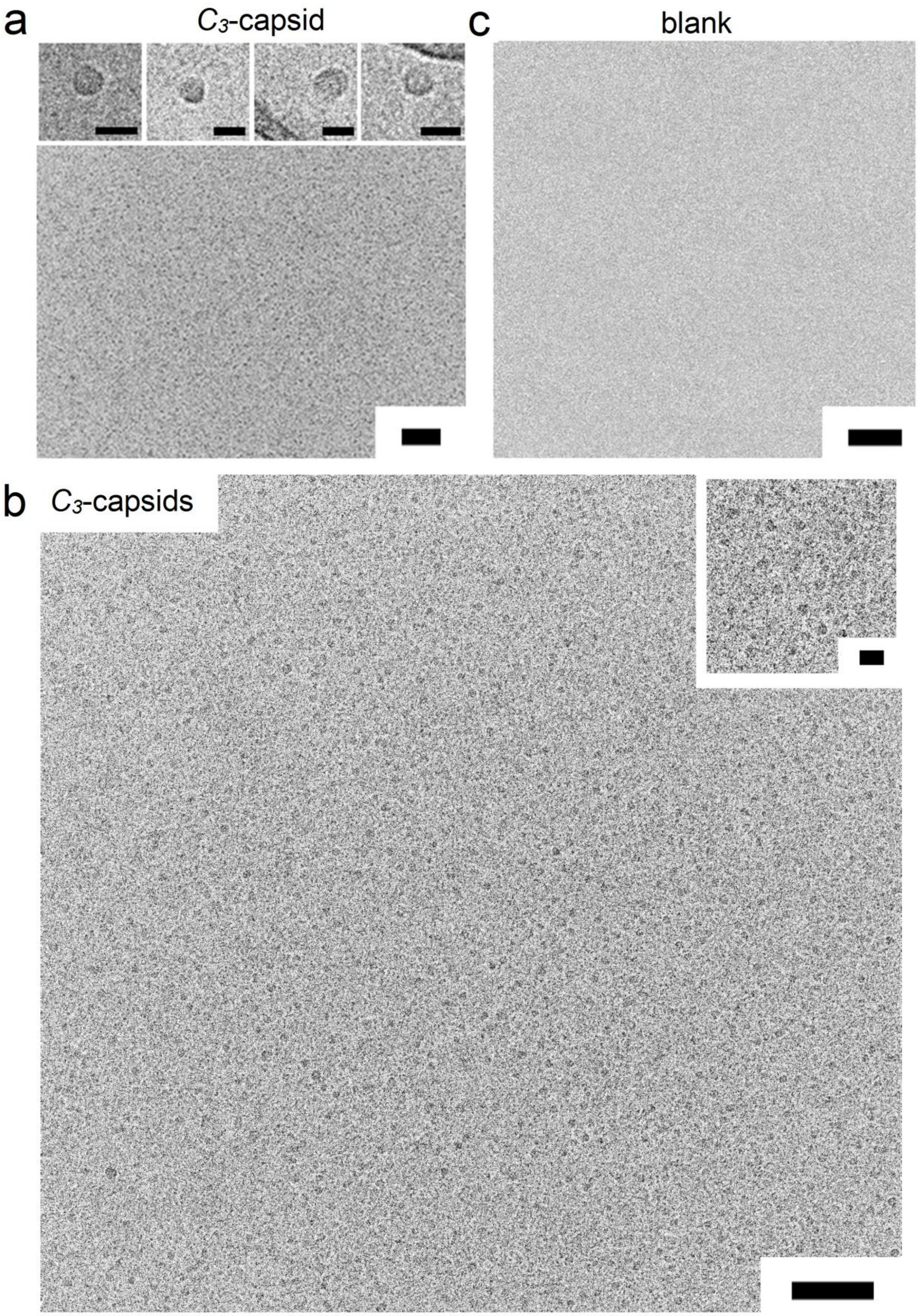

Supplementary Figure 6. Morphology of C_3-capsids. **a** Cryo electron micrographs of assembled C_3-capsids. Scale bars are 100 nm, and 20 nm for the inset images. **b** Larger area cryo electron micrographs of assembled C_3-capsids. Scale bars are 100 nm, and 25 nm for the inset image. **c** Cryo electron micrographs of blank control samples. Scale bar is 100 nm.

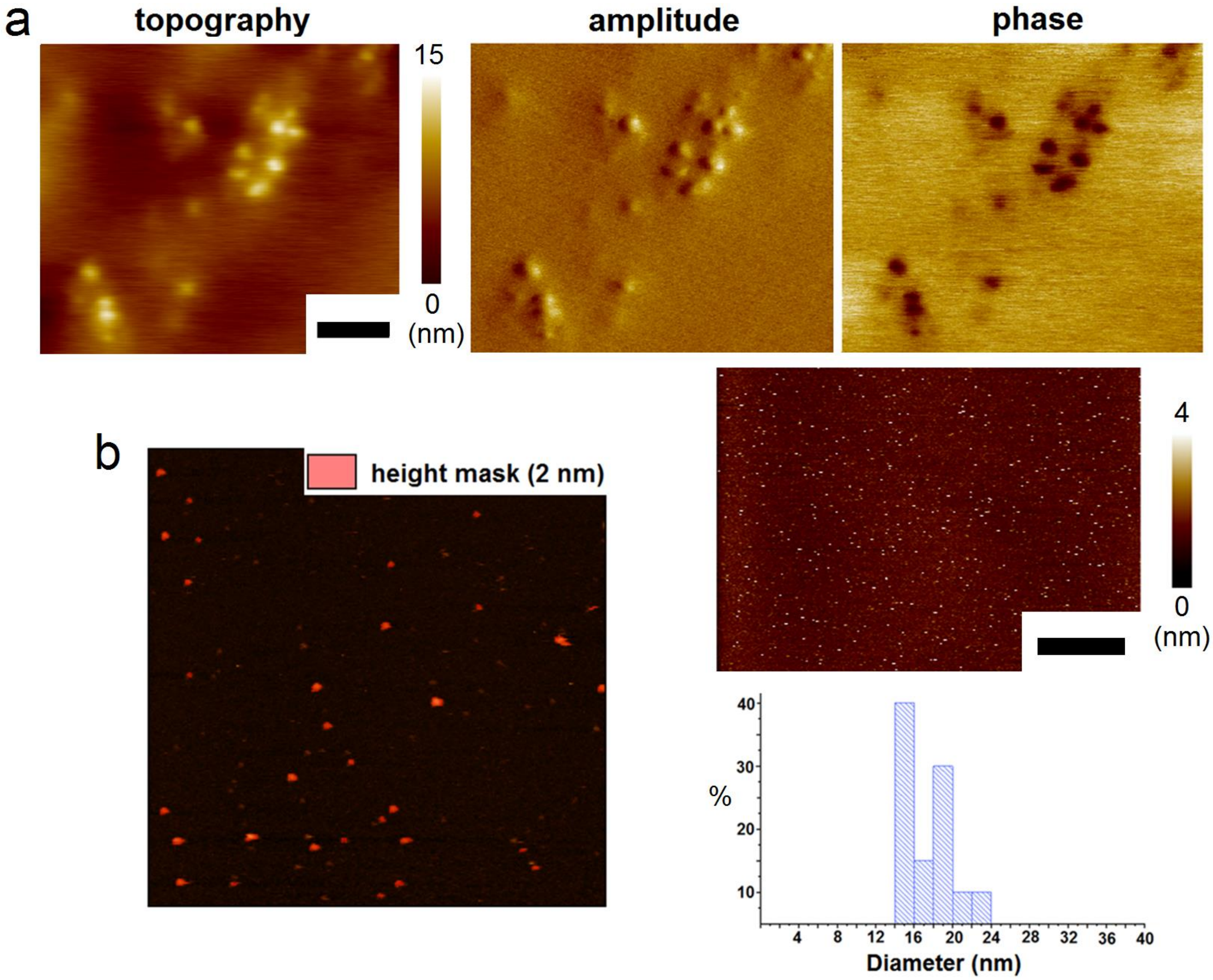

Supplementary Figure 7. Morphology of C_3-capsids. **a** In-air AFM images of assembled capsids. Scale bar is 100 nm. **b** In-water AFM analysis of assembled capsids. Scale bar is 1 μm.

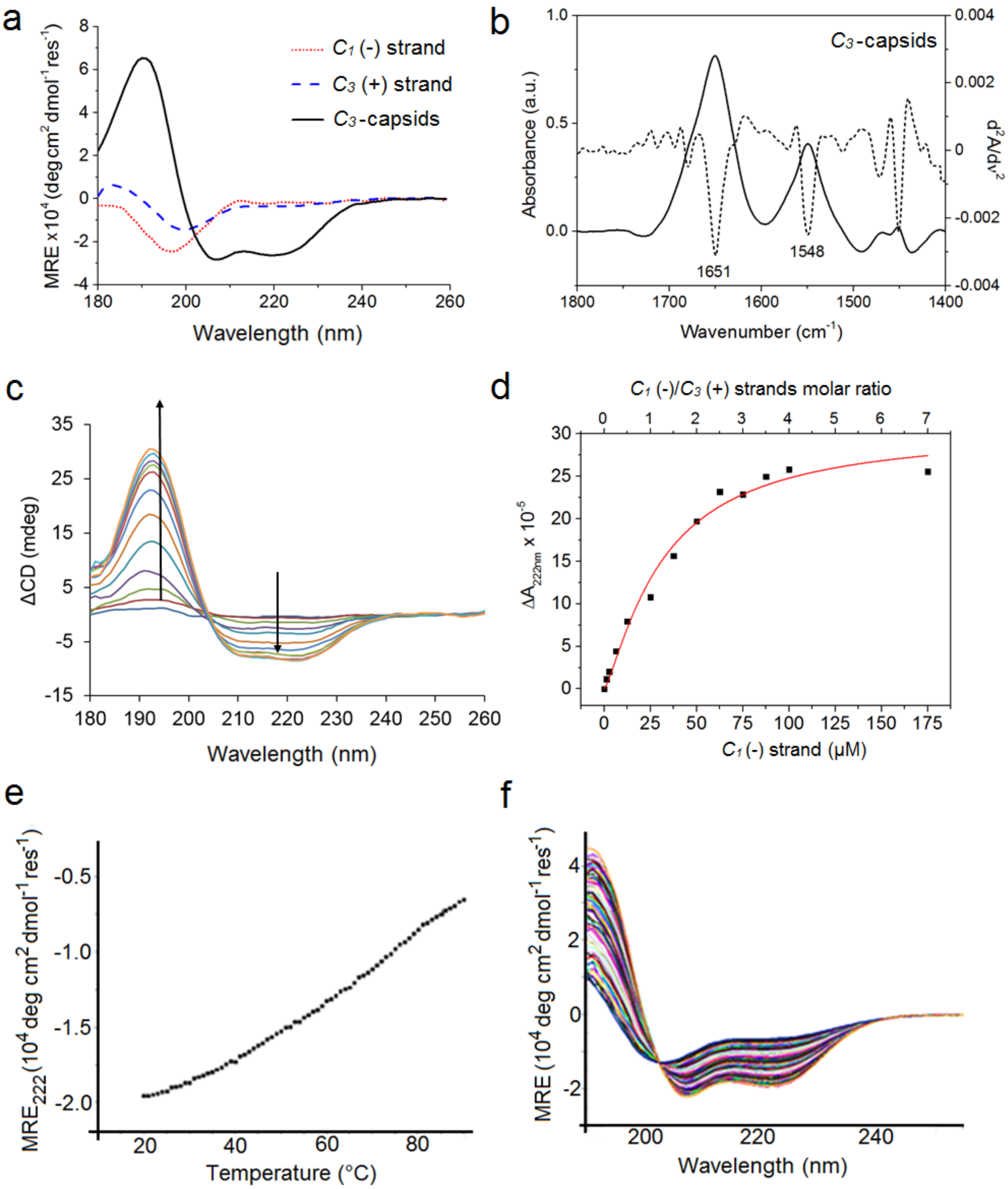

Supplementary Figure 8. Folding of C_3-capsids. **a** CD spectra for C_1 (-) strand (red dotted), C_3 (+) strand (blue dashed line) and their 3:1 molar mixture – assembled C_3-capsids (black line). **b** A representative FT-IR spectrum (solid line) and its second derivative (dotted line) for the assembled C_3-capsids. Key: 100 µM peptide (total), 10 mM MOPS, 5 mM TCEP, pH 7.4. **c** Synchrotron radiation CD spectra for C_1 (-) strand titrated into C_3 (+) strand (25 µM) and **d** plotted as ΔA at 222 nm versus the concentration of C_1 (-) strand and its molar ratio to C_3 (+) strand. Arrows in **c** indicate the increased concentration of C_1 (-) strand. The data in **d** was fitted into the Hill equation to give K_D of 32 ± 5.6 µM (R^2 = *0.98*). **e** Thermal unfolding curve for C_3-capsids as a function of temperature at 222 nm. **f** CD spectra following the thermal unfolding with individual spectra recorded every 1°C. Note the isodichroic point at 202 nm.

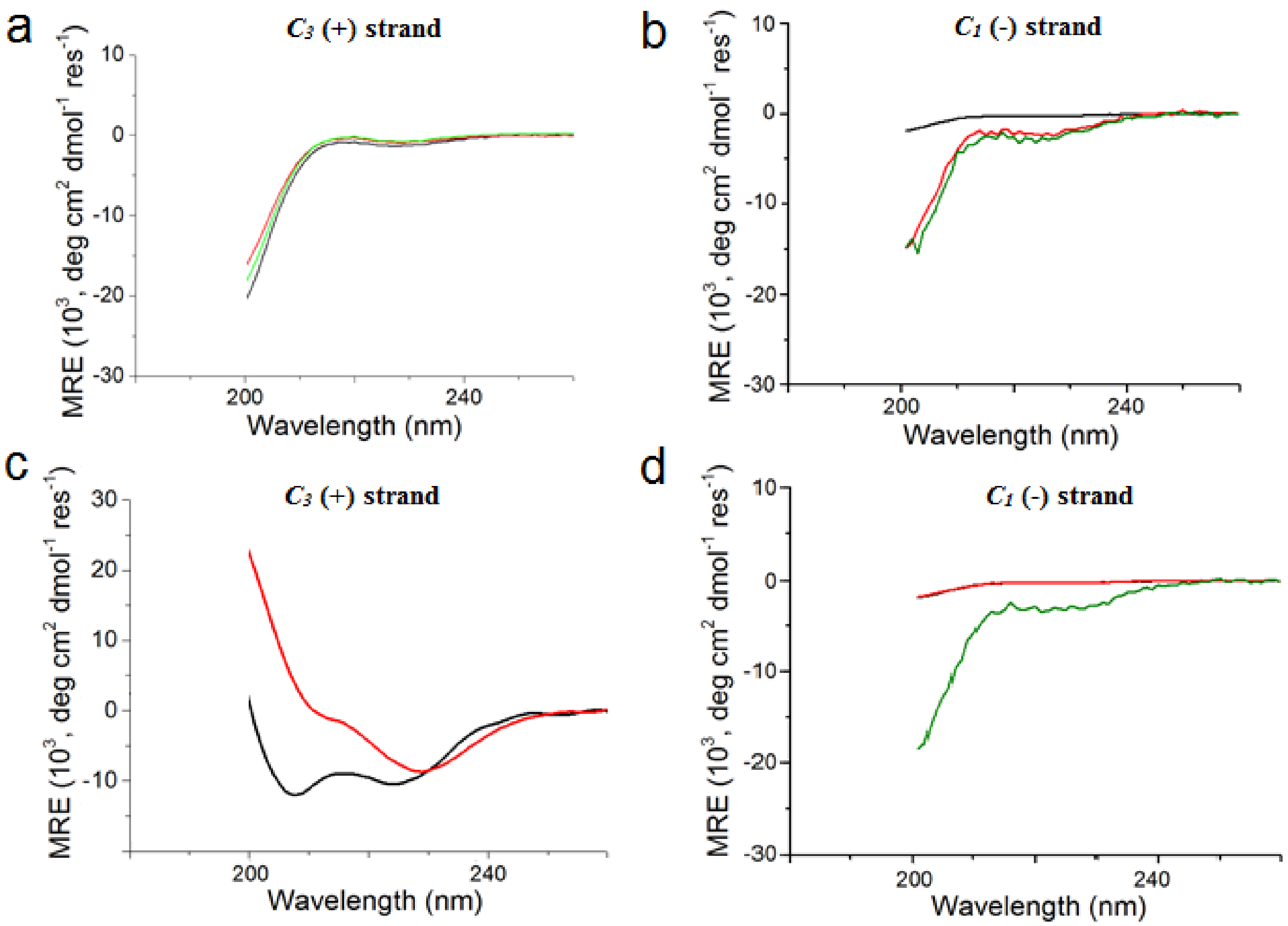

Supplementary Figure 9. Folding of the capsid components in AUVs and ZUVs at L/P 100 (50 µM total peptide). CD spectra for **a** C_3 (+) strand and **b** C_1 (-) strand in 10 mM MOPS, pH 7.4 (black) and in DLPC (red) and POPC (green). **c** CD spectra for C_3 (+) strand in DLPC/DLPG (3:1, molar ratio) (red) and POPC/POPG (3:1, molar ratio) (black). **d** C_1 (-) strand in the buffer (black), DLPC/DLPG (3:1, molar ratio) (red) and POPC/POPG (3:1, molar ratio) (green).

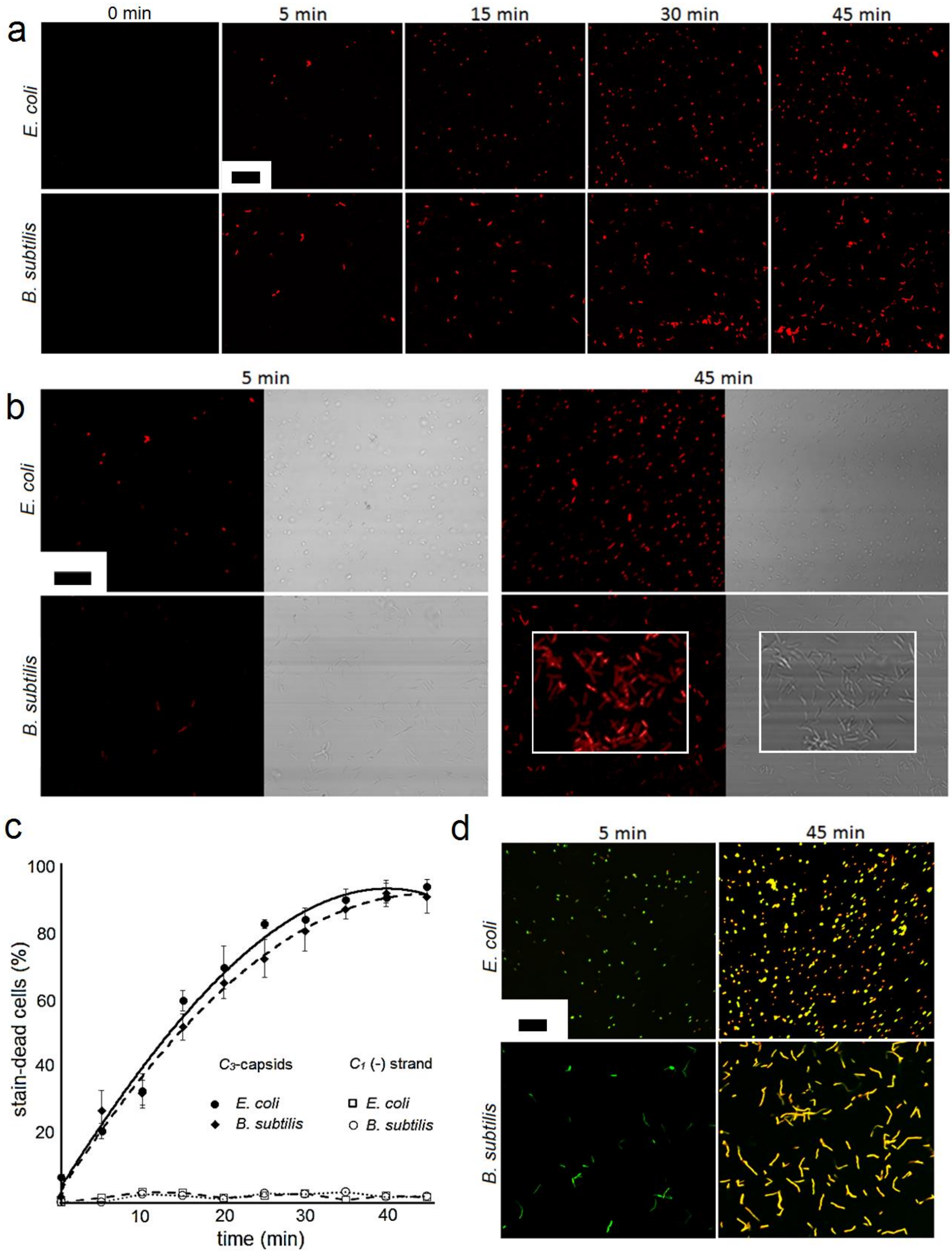

Supplementary Figure 10. Antimicrobial kinetics of C_3-capsids. **a** Fluorescence microscopy images taken at different time points for bacteria incubated with C_3-capsids at the MICs (3 μM) and stained with propidium iodide (PI). **b** PI-fluorescence and corresponding contrast images, together with high-magnification inset image, shown only for 5 min and 45 min for clarity. **c** Average numbers of stain-dead cells as a function of time after subtracting background numbers (buffer), for cells incubated with C_3-capsids and with the C_1 (-) strand as a negative control. The data represent mean values ± s. d. **d** Overlaid fluorescence microscopy images of bacterial cells following dual-colour assay (LIVE/DEAD® BacLight™) – SYTO®9 (green) and PI (red) monitored at 515 nm and 625 nm, respectively. Red and merged (yellow) stains indicate lysed bacteria. Scale bars are 30 μm.

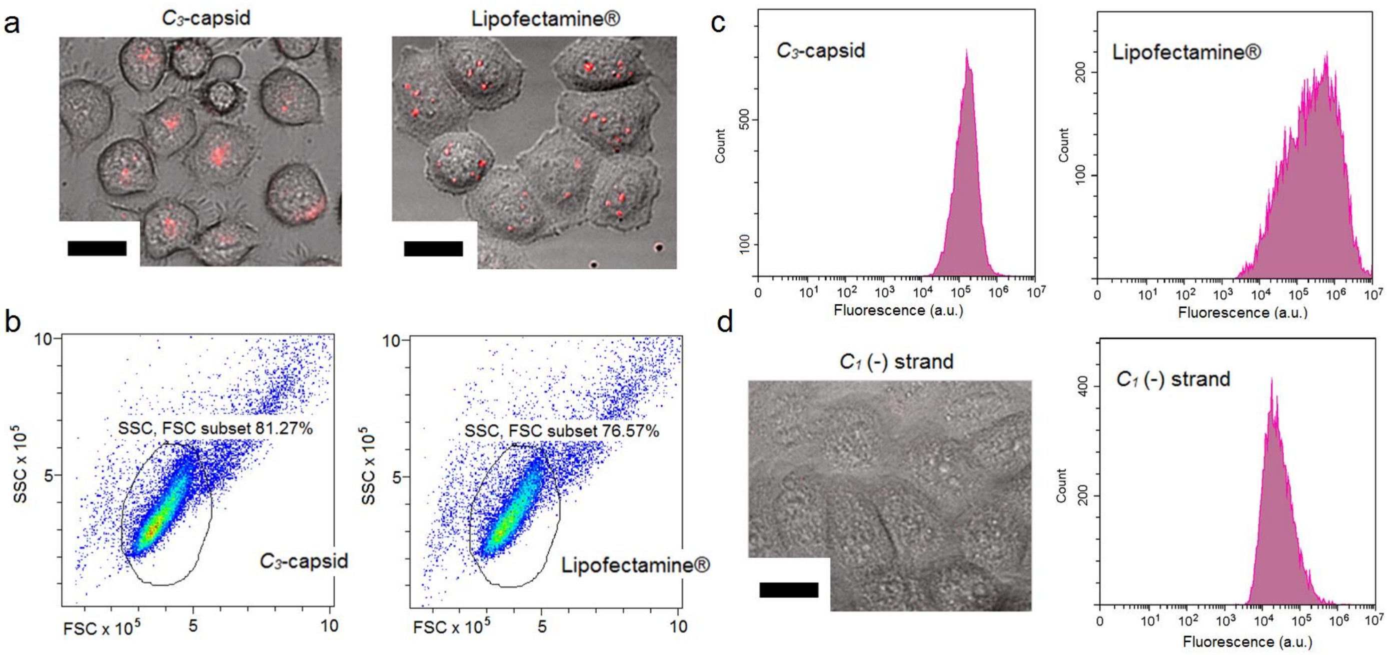

Supplementary Figure 11. C_3-capsids infecting human cells. **a** Overlaid confocal fluorescence and bright-field images of HeLa cells incubated for 4 h at 37°C with AF647-siRNA (red) pre-assembled with C_3-capsids at a P/N charge ratio of 8:1 and with Lipofectamine® RNAiMax as per the proprietary protocols. Scale bars are 25 µm. **b** Flow cytometry charts of cell population gating: at least 10^5 events were gated (circled in the charts) from each $\geq 10^6$ subset measured for each sample by forward scatter and side scatter (X and Y axis, respectively) on the 488-nm laser **c** to extract and analyse 10^4 single viable cells. **d** Overlaid images and flow cytometry chart of HeLa cells incubated for 4 h at 37°C with AF647-siRNA pre-incubated with C_1 (-) strand, as a negative control. Scale bar is 25 µm.
