# Supplementary material for: Antimicrobial peptide capsids of de novo design
Source: Nat Commun. 2017 Dec 22;8:2263. doi: 10.1038/s41467-017-02475-3 (PMC5741663; doi:10.1038/s41467-017-02475-3)
Supplement: Supplementary file 2 — Description of Additional Supplementary Files [file 41467_2017_2475_MOESM2_ESM.pdf]

Description of Additional Supplementary Files

File Name: Supplementary Movie 1
Description: Time-lapse in-water AFM imaging of supported lipid bilayers treated with C_3-capsides (~ 3 µM total peptide). The full movie is shown here, visualising complete bilayer poration at 13 seconds per frame. The time-stamp corresponds to the middle line of each AFM scan and refers to approximate time elapsed since capsid injection. Height scale as in Figure 5.
